# Effect of Fluence Rate on Tumor Tissue Damage in IRDye700DX‐Based Photoimmunotherapy

**DOI:** 10.1002/cam4.71143

**Published:** 2025-08-19

**Authors:** Susumu Yamashita, Miho Kojima, Nobuhiko Onda, Makoto Shibutani

**Affiliations:** ^1^ Laboratory of Veterinary Pathology, Division of Animal Life Science Institute of Agriculture, Tokyo University of Agriculture and Technology Tokyo Japan; ^2^ Medical Evaluation Engineering, Olympus Medical Systems Corp. Tokyo Japan; ^3^ Institute of Global Innovation Research, Tokyo University of Agriculture and Technology Tokyo Japan

**Keywords:** antibody–photosensitizer conjugate, fluence rate, IRDye700DX, light dose, near‐infrared photoimmunotherapy

## Abstract

**Objective:**

IRDye700DX‐based photoimmunotherapy (IR700‐based PIT) is a newly developed cancer phototherapy using an antibody–photosensitizer conjugate (APC). The APC for IR700‐based PIT (mAb‐IR700) bound to target molecules on the membrane of cancer cells causes rapid cell necrosis following light irradiation. It is thus reasonable to consider that the light irradiation in IR700‐based PIT influences its therapeutic effect. However, the relationship between the therapeutic effect and light irradiation conditions has remained unclear. This study aimed to investigate the influence of the light dose and fluence rate on IR700‐based PIT efficacy.

**Methods:**

We first examined the effect on cytotoxicity in vitro. Epidermal growth factor receptor (EGFR)‐overexpressing A431 tumor cells were incubated with an mAb‐IR700 targeting EGFR and exposed to light. Next, the effect on tumor tissue damage was examined in vivo. A431 cells and two human epidermal growth factor receptor 2‐overexpressing tumor cell lines were subcutaneously grafted into BALB/c nude mice. The mice were exposed to light 1 day after mAb‐IR700 injection.

**Results:**

The number of propidium iodide‐positive dead cells in vitro increased in a light dose‐dependent manner but was not influenced by fluence rate. One day after light irradiation in vivo revealed that the tumor tissue damage increased in a light dose‐dependent manner but decreased with increasing fluence rate in the three tumor‐grafted animal models. Furthermore, tumor growth inhibition data after 24 days from IR700‐based PIT was consistent with the acute tissue‐damage data.

**Conclusion:**

This study demonstrates that fluence rate as well as light dose impacts in vivo anti‐tumor effect, and the effects on tumor microenvironment might be responsible for the discrepancy from in vitro results.

## Graphical abstract

The effects of the light dose and fluence rate of NIR‐PIT on its efficacy were analyzed. The obtained results showed that light dose influenced the level of cancer cell cytotoxicity in vitro and in vivo, while the fluence rate had no effect in vitro, but had a negative association with NIR‐PIT efficacy in vivo.

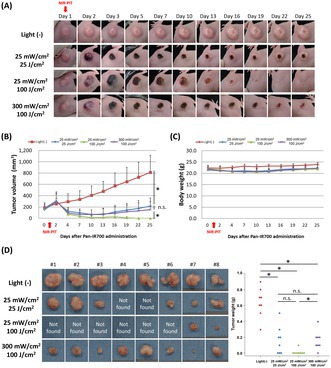

**Abbreviations**
APC: antibody–photosensitizer conjugate
EGFR: epidermal growth factor receptor
HER2: human epidermal growth factor receptor 2
Hoechst: Hoechst 33,342
IR700: IRDye700DX
LDH: lactate dehydrogenase
NADH‐TR: nicotinamide adenine dinucleotide‐tetrazolium reductase
PI: propidium iodide
PIT: photoimmunotherapy
ROI: region of interest
Tra‐IR700: trastuzumab‐IRDye700DX

## 1 Introduction

Photoimmunotherapy (PIT) is a cancer therapy using a combination of antibody–photosensitizer conjugates (APCs) and light exposure. PIT is one of the molecular targeted therapies, and it has historically been applied to improve the delivery of cell type non‐specific photosensitizers to target tumor cells, such as hematoporphyrin, benzoporphyrin derivative, and chlorin‐e6 [1, 2, 3]. Recently, a novel form of PIT which employs a phthalocyanine dye, IRDye700DX (IR700), as a photosensitizer has been developed [4, 5, 6]. An APC for IR700‐based PIT (mAb‐IR700) binds to target molecules on the cancer cell membrane and forms a water‐insoluble aggregation after exposure to NIR light at an excitation wavelength of approximately 690 nm, resulting in irreparable membrane damage and rapid cell necrosis [7]. Historically, the efficacy of PIT has been shown based on the generation of reactive oxygen species (ROS) by photosensitizers [8]. Thus, IR700‐based PIT is considered to be unique in terms of the induction of physical stress in the target cell membrane, resulting in cell disruption. IR700‐based PIT is applicable to the targeting of various cancer antigens by changing the antibodies employed. In several pre‐clinical studies, it has been reported that IR700‐based PIT using a variety of different mAb‐IR700 exerts anti‐tumor effects in various cancers [9]. In a clinical context, a global Phase 3 trial of ASP‐1929, an anti‐epidermal growth factor receptor (EGFR) antibody IR700 conjugate for recurrent or inoperable head and neck cancer patients, is currently ongoing (NCT03769506). In Japan, ASP‐1929 (Akalux; Rakuten Medical Inc., San Diego, CA, USA) was approved for clinical use in September 2020 for the first time globally, combined with a 690 nm laser system (BioBlade; Rakuten Medical Inc.) [10].

Several parameters have been reported to influence the efficacy of IR700‐based PIT in pre‐clinical study [11, 12, 13, 14, 15, 16]. Among them, the relationship between light dose and therapeutic effect has been well studied, and it has been shown that the anti‐tumor effect of IR700‐based PIT is dependent on the light dose in vitro and in vivo [17, 18, 19, 20, 21]. Thus, light irradiation conditions may have a significant impact on the efficacy of IR700‐based PIT. Fluence rate is considered to be a major light irradiation parameter besides light dose. It is believed that light irradiation at a high fluence rate is ideal because the light dose per unit time is increased and the treatment time is reduced. Until now, there is one report investigating the relationship between fluence rate and the efficacy of IR700‐based PIT [22]; however, that study provides data limited to one model. Because IR700‐based PIT can be applied to many kinds of tumors, it is thus important to investigate the influence of major light irradiation conditions, especially fluence rate, on the anti‐tumor effect of IR700‐based PIT to maximize efficacy and optimize clinical outcomes.

In the present study, we first investigated the influence of light irradiation dose and fluence rate on the efficacy of IR700‐based PIT in vitro. Then, we further examined the impact of the light irradiation conditions of IR700‐based PIT in vivo using three mouse xenograft models.

## 2 Materials and Methods

### 2.1 Reagents

A water‐soluble, silica‐phthalocyanine derivative, IRDye700DX NHS ester, was obtained from LICOR Biosciences (Lincoln, NE, USA). Panitumumab (Vectibix), a fully humanized IgG2 mAb directed against human EGFR, was purchased from Takeda Pharmaceutical Co. Ltd. (Osaka, Japan). Trastuzumab (Herceptin), a 95% humanized IgG1 mAb against the extracellular domain of human epidermal growth factor receptor 2 (HER2), was purchased from Chugai Pharmaceutical (Tokyo, Japan). The conjugation of IR700 with antibodies was performed in accordance with our previous report [23].

### 2.2 Cell Culture

The three human cancer cell lines used in this study are described in Table S1. The human epidermal carcinoma cell line A431 was used as an EGFR‐positive cell line, and the human gastric carcinoma cell line NCI‐N87 and the human breast ductal carcinoma cell line BT‐474 were used as human epidermal growth factor receptor 2 (HER2)‐positive cell lines, with reference to previous IR700‐based PIT studies [4, 23, 24].

### 2.3 Flow Cytometry

Cells that had reached approximately 80% confluence in T‐25 flasks were incubated with 10 μg/mL of mAb‐IR700 for 1 h. Then, the cells were washed twice with PBS, detached by trypsin, and collected by centrifugation. The fluorescence of mAb‐IR700 bound to cells was measured for 10,000 cells using a CytoFLEX flow cytometer equipped with a 638 nm laser, a 712/25 nm band‐pass filter (Beckman Coulter, Brea, CA, USA), and CytExpert software (Beckman Coulter).

### 2.4 Fluorescence Imaging Systems

Live‐cell images were captured using a fluorescence inverted microscope (IX‐83; Olympus Corporation, Tokyo, Japan) or a fluorescence virtual slide scanning system (VS120‐FL; Olympus Corporation). VS120‐FL was also used for tissue section imaging. Macroscopic images of mice were captured using an in‐house NIR fluorescence imaging system consisting of excitation light provided by a light emitting diode (LED) at a peak of 660 nm (SMBB660‐1100‐02; Ushio Opto Semiconductors, Tokyo, Japan) and a monochrome camera (GS3‐U3‐15S5M‐C; Point Grey Research, Vancouver, BC, Canada) equipped with a 692‐nm long‐pass filter (FF01‐692/LP‐25‐D; Semrock, Rochester, NY, USA). All fluorescence images were converted to pseudo‐colors using cellSens Dimension software (Olympus Corporation) or ImageJ software (NIH, Bethesda, MD, USA). The analysis of fluorescence images was performed using ImageJ software.

### 2.5 Live‐Cell Imaging

Cells were seeded onto 8‐well chamber slides and incubated for 24 h. Culture medium was replaced with fresh medium containing 10 μg/mL of mAb‐IR700 and incubated for 1 h or 24 h. Then, cells were washed twice with PBS, and nuclei were stained using Hoechst 33,342 (Thermo Fisher Scientific Inc., Waltham, MA, USA). To determine the intracellular localization of mAb‐IR700, the cells were co‐stained with the organelle markers listed in Table S2.

### 2.6 In Vitro IR700‐Based PIT

Cells were seeded onto 96‐well plates and incubated for 24 h. Culture medium was replaced with fresh medium containing 10 μg/mL of mAb‐IR700 and incubated for 1 h or 24 h. Then, the cells were irradiated with NIR light using a 690 nm continuous‐wave laser (BWF1‐690‐300‐E; B&W TEK, Newark, DE, USA). Cells were irradiated at 1, 2.5, 5, and 10 J/cm^2^ using all fluence rate settings (12.5, 25, 50, and 100 mW/cm^2^). The fluence rate was monitored with a radiometer (PD300‐BB‐50 mW; OPHIR Photonics, Jerusalem, Israel).

### 2.7 In Vitro Cytotoxicity Assay

One day after light irradiation, morphological changes of cells were observed by phase‐contrast microscopy. The cytotoxic effects of IR700‐based PIT on cultured cells were also determined by Hoechst/propidium iodide (PI) double‐staining and the lactate dehydrogenase (LDH) cytotoxicity assay. For Hoechst/PI double‐staining, 1 day after IR700‐based PIT, cells were incubated with fresh medium containing PI (500 nM; Thermo Fisher Scientific Inc.) and Hoechst (5 μg/mL) for 10 min at room temperature and washed twice with PBS. Fluorescence images were captured in the same region. The LDH assay was performed on culture supernatants 1 day after light irradiation using Cytotoxicity LDH Assay Kit‐WST (Dojin Chemicals, Kumamoto, Japan). Absorbance at 490 nm was measured by a microplate reader (Nivo S; PerkinElmer, Waltham, MA, USA) and the cytotoxicity rate was calculated in accordance with the manufacturer's protocol.

### 2.8 Animal Model

Nude mice (BALB/c‐nu, female, 4 weeks old) were purchased from the Jackson Laboratory Japan Inc. (Yokohama, Japan) and acclimated for 1 week. A431, NCI‐N87, and BT‐474 cells were suspended in growth medium; for the BT‐474 cells, the suspension was subsequently mixed with Matrigel (356237; Corning, New York, NY, USA) at a 1:1 ratio. The mice were subcutaneously inoculated with 5 × 10^6^ A431 cells, 5 × 10^6^ NCI‐N87 cells, or 1 × 10^7^ BT‐474 cells. Tumor volume was calculated using the following formula: tumor volume = 1/2 × length × width [2]. Tumors were studied after they reached a volume of at least 20 mm^3^.

### 2.9 In Vivo and Cellular Distribution Analysis of Administered mAb‐IR700

Mice were administered mAb‐IR700 at 300 μg/mouse intravenously via the retro‐orbital vein. In vivo fluorescence and white light images were obtained at each time point. Regions of interest (ROIs) were placed on the tumor and left dorsum region (nontumorous region opposite the tumor region as background) in the fluorescence images with the white light image as a reference, and the mean fluorescence intensity of each ROI was measured. Mice were euthanized by exsanguination under deep isoflurane anesthesia 1 day after the administration. Resected tumor tissues were snap‐frozen in liquid nitrogen. Unfixed frozen sections were subjected to observation for the cellular distribution of administered mAb‐IR700 and then fixed with methanol. Fixed frozen sections were immunostained with the primary and secondary antibodies listed in Table S3, followed by counterstaining with DAPI (Thermo Fisher Scientific Inc.).

### 2.10 In Vivo IR700‐Based PIT

In the evaluation of the immediate effects of IR700‐based PIT on xenografted tumor tissues after light irradiation, mice were randomized into six groups: (i) Control, light (−); (ii) IR700‐based PIT at 25 mW/cm^2^ and 25 J/cm^2^; (iii) IR700‐based PIT at 25 mW/cm^2^ and 50 J/cm^2^; (iv) IR700‐based PIT at 25 mW/cm^2^ and 100 J/cm^2^; (v) IR700‐based PIT at 100 mW/cm^2^ and 100 J/cm^2^; and (vi) IR700‐based PIT at 300 mW/cm^2^ and 100 J/cm^2^. In the evaluation of the long‐term effects of IR700‐based PIT on xenografted tumor tissues after light irradiation, A431 tumor‐bearing mice with a tumor volume of approximately 200 mm^3^ were selected and randomly divided into four groups: (i), (ii), (iv), and (vi), as defined in the item on evaluation of immediate effects. Details regarding the number of animals in each group for the A431, NCI‐N87, and BT‐474 xenograft models are shown in Table S4. Light irradiation was performed with a 690 nm continuous‐wave laser (ML7710‐690; Modulight, Tampere, Finland) under isoflurane anesthesia 1 day after mAb‐IR700 injection. To follow up the therapeutic effect after IR700‐based PIT, tumor size and body weight were measured three times a week, and irradiated sites were photographed with a digital camera (TG‐Tracker, Olympus Corporation) before light irradiation and after the irradiation for up to 24 days. Mice were euthanized by exsanguination via the abdominal aorta under deep isoflurane anesthesia 1 or 24 days after IR700‐based PIT, and tumor tissues were harvested for photography and weight measurements.

### 2.11 Histological Analysis

Harvested tumor tissues were divided into two pieces. One was fixed with 4% paraformaldehyde overnight at 4°C, and the other was snap‐frozen in liquid nitrogen. Paraffin‐embedded sections were stained with H&E. We measured the damaged area of neoplastic cells characterized by necrotic changes showing nuclear pyknosis or fragmentation, eosinophilic cytoplasm, and microhemorrhages in H&E‐stained tumor tissue sections. For histopathological evaluation, acellular regions in H&E‐stained tumor tissue sections were excluded from measuring the damaged area. Regarding the A431 tumors, the cornified layer, consisting of degenerated keratinocytes, was also excluded from the measurement of the damaged area. Damaged areas larger than 100 μm were traced in H&E images while blinded to the treatment conditions using Photoshop Elements 2019 (Adobe Systems, San Jose, CA, USA). Tumor damage was calculated as the percentage of the damaged area relative to the total tumor area. Nicotinamide adenine dinucleotide‐tetrazolium reductase (NADH‐TR) staining was performed to identify tumor tissue damage in parallel with the assessment of H&E staining. Staining solution was prepared by adding 0.024 g of β‐NADH and 0.03 g of nitro blue tetrazolium to 30 mL of Tris–HCl buffer (pH 7.4). Cryosections were placed in staining solution for 30 min at 37°C, and then washed and mounted.

### 2.12 Statistical Analysis

Numerical data are presented as the mean ± SD. Numerical data were assessed using Tukey's test after verification of the homogeneity of the variances by Bartlett's test. The Steel–Dwass test was used for heterogeneous data; *p*‐values less than 0.05 were considered statistically significant.

## 3 Results

**FIGURE 1:**
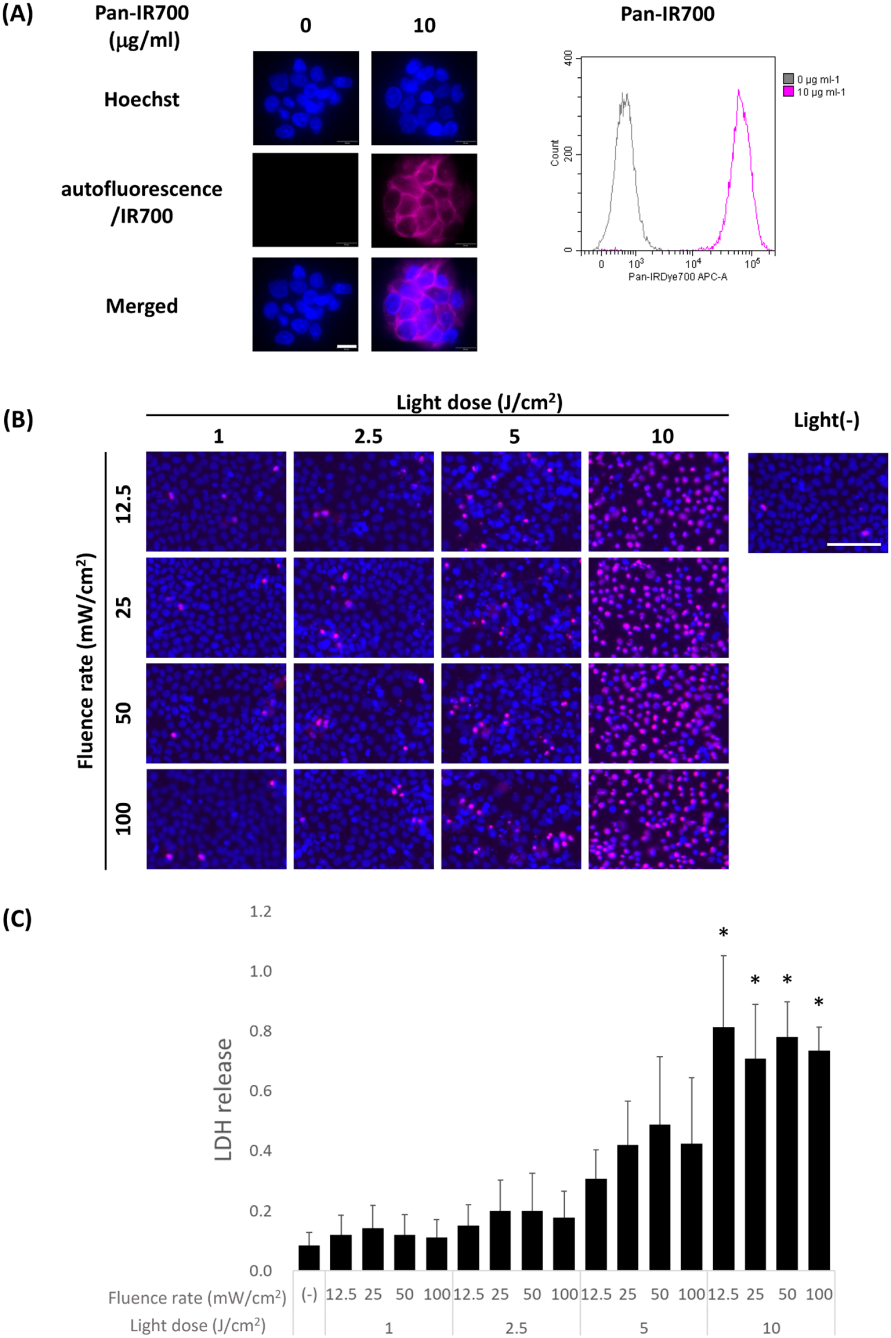
Influence of light irradiation conditions on efficacy of IRDye700DX‐based photoimmunotherapy (IR700‐based PIT) in vitro. (A) Responses of A431 cells to synthesized panitumumab‐IRDye700DX (Pan‐IR700). Fluorescence images and flow cytometry analysis of A431 cells incubated with or without Pan‐IR700. Scale bar = 20 μm. (B) Hoechst 333,42 (blue) and propidium iodide (red) double‐stained images 1 day after IR700‐based PIT in A431 cells exposed to various light irradiation conditions. Scale bar = 100 μm. (C) Lactate dehydrogenase (LDH) assay 1 day after IR700‐based PIT in A431 cells exposed to various light irradiation conditions. Data are presented as mean ± SD (*n* = 3, Tukey's test; * statistically significant difference from control group at *p* < 0.05).

The binding capability of synthesized Pan‐IR700 in the EGFR‐positive A431 cell line was analyzed by fluorescence microscopy and flow cytometry. A431 cells without any treatment did not show an autofluorescence signal under Pan‐IR700 observation conditions. After incubation with Pan‐IR700 for 1 h, A431 cells showed Pan‐IR700 fluorescence on their surface (Figure 1A). Then, A431 cells incubated with Pan‐IR700 for 1 h were subjected to light irradiation, and the influence of light dose and fluence rate on the cytotoxicity of IR700‐based PIT was examined using Hoechst/PI double‐staining. The number of PI‐positive dead cells increased in a light dose‐dependent manner, but the number of dead cells did not differ in a manner dependent on the fluence rate (Figure 1B). No toxicity was induced by Pan‐IR700 incubation alone. Morphological changes, such as cell swelling and rupturing, were observed in correlation with an increase in the number of PI‐positive cells (Figure S1). The influence of light irradiation conditions on IR700‐based PIT cytotoxicity was demonstrated by LDH release in accordance with the PI‐positive fluorescence signals (Figure 1C). LDH release was significantly increased at 10 J/cm^2^ irrespective of the fluence rate. Meanwhile, the fluence rate did not significantly affect LDH release at any irradiation dose. The cellular distribution of Pan‐IR700 was analyzed over time. IR700 fluorescence was observed at the cell membrane after 1 h of Pan‐IR700 incubation, while at 24 h after incubation the fluorescence was observed mainly in lysosomes (Figure S2A). The effect of light dose and fluence rate on the cytotoxicity of IR700‐based PIT in A431 cells incubated with Pan‐IR700 for 24 h was in accordance with IR700‐based PIT at 1 h after Pan‐IR700 incubation, showing an increase in a light dose‐dependent manner and was not affected by fluence rate (Figure S2B,C).

**FIGURE 2:**
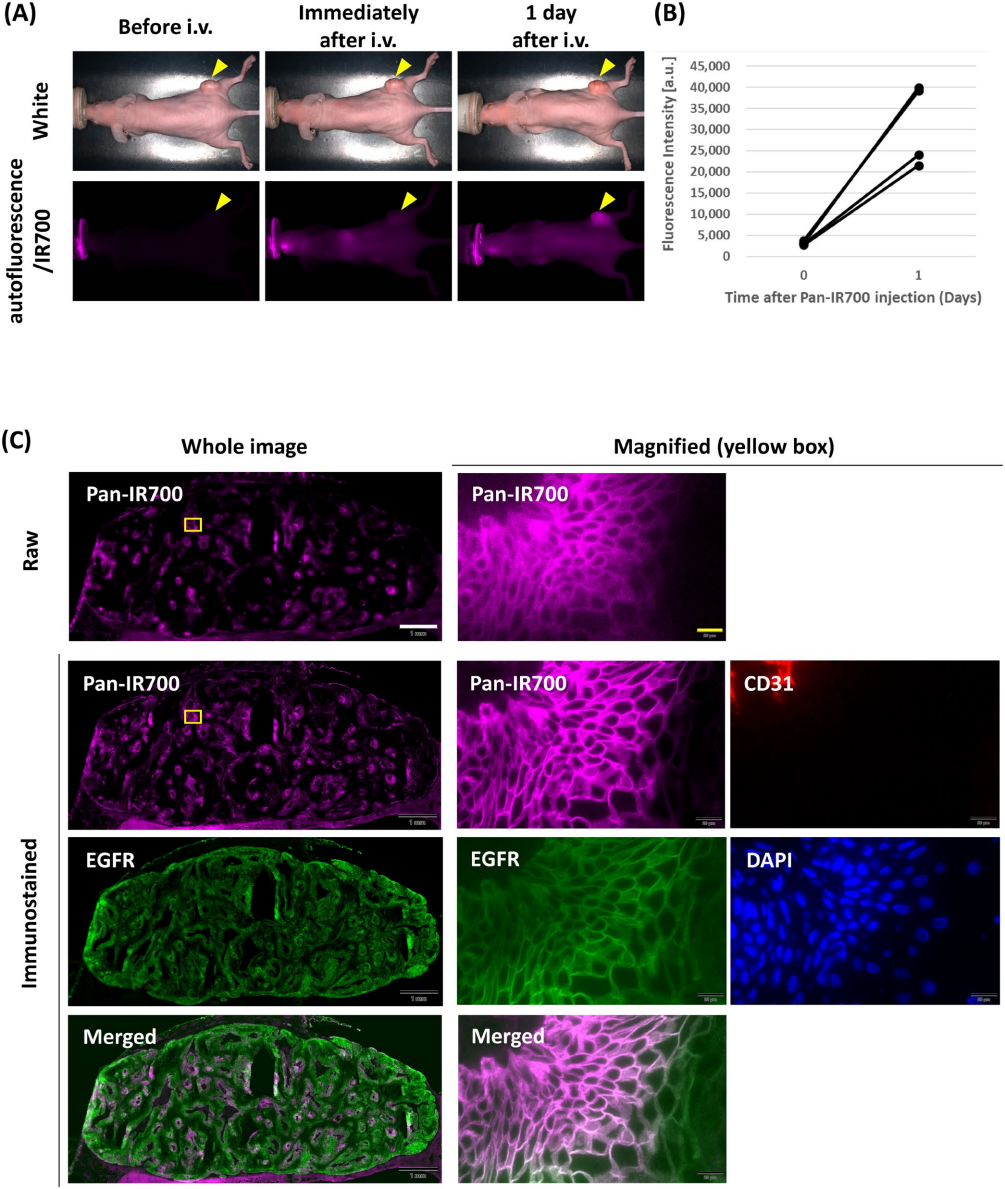
Fluorescence imaging of administered Pan‐IR700 in A431 tumors. (A) Time‐course imaging of Pan‐IR700 fluorescence in A431 tumor‐bearing mice (right dorsum as shown by yellow arrow). Before i.v. image indicates the autofluorescence signal. (B) Changes in the fluorescence signal intensity by the administration of Pan‐IR700 (*n* = 4). (C) Tissue and cellular distribution of Pan‐IR700 in A431 tumors 1 day after injection. The top panel shows fluorescence image of administered Pan‐IR700 (magenta) in unfixed frozen sections. The panels from the second row onwards are fluorescent immunostaining images of the frozen sections. The colors of each image correspond to the stained molecules as follows: Panitumumab (magenta), epidermal growth factor receptor (green), cluster of differentiation 31 (red), and DAPI (blue). The merged image of panitumumab and epidermal growth factor receptor was generated to show merged signals as white in color. White scale bar = 1 mm. Yellow scale bar = 20 μm.

A431 tumor‐bearing mice showed high EGFR immunoreactivity within the tumor tissues, especially in the basal cell layer (Figure S3). Before Pan‐IR700 administration, A431 tumor‐bearing mice did not show any autofluorescence signal under Pan‐IR700 observation conditions. In vivo imaging showed that the Pan‐IR700 fluorescence in A431 tumors was increased 1 day after administration (Figure 2A,B). The fluorescence signal of Pan‐IR700 was heterogeneously distributed throughout the tumor tissues. The distribution pattern of intravenously injected Pan‐IR700 overlapped that of tumor cells, as visualized by EGFR immunofluorescence staining, and overlapped that of EGFR immunofluorescence staining (Figure 2C). The intensity of the signals was highest around the CD31‐positive vascular endothelial cells and weakened with increasing distance from the vasculature. At the cellular level, the Pan‐IR700 fluorescence signal was localized at the surface of tumor cells (Figure 2C). In contrast, A431 tumors without Pan‐IR700 injection did not show any autofluorescence in tumor sections (Figure S4A). In the colocalization analysis of EGFR and Pan‐IR700 fluorescence signals employing the Pearson's R value and Mander's tM1, these values were increased in the tumors with Pan‐IR700 injection compared to the tumors without Pan‐IR700 injection (Figure S4B). Since the in vitro cytotoxicity of IR700‐based PIT was caused by light irradiation at the time the Pan‐IR700 fluorescence was observed on the tumor cell surface, light irradiation was planned 1 day after Pan‐IR700 administration in subsequent in vivo IR700‐based PIT studies.

**FIGURE 3:**
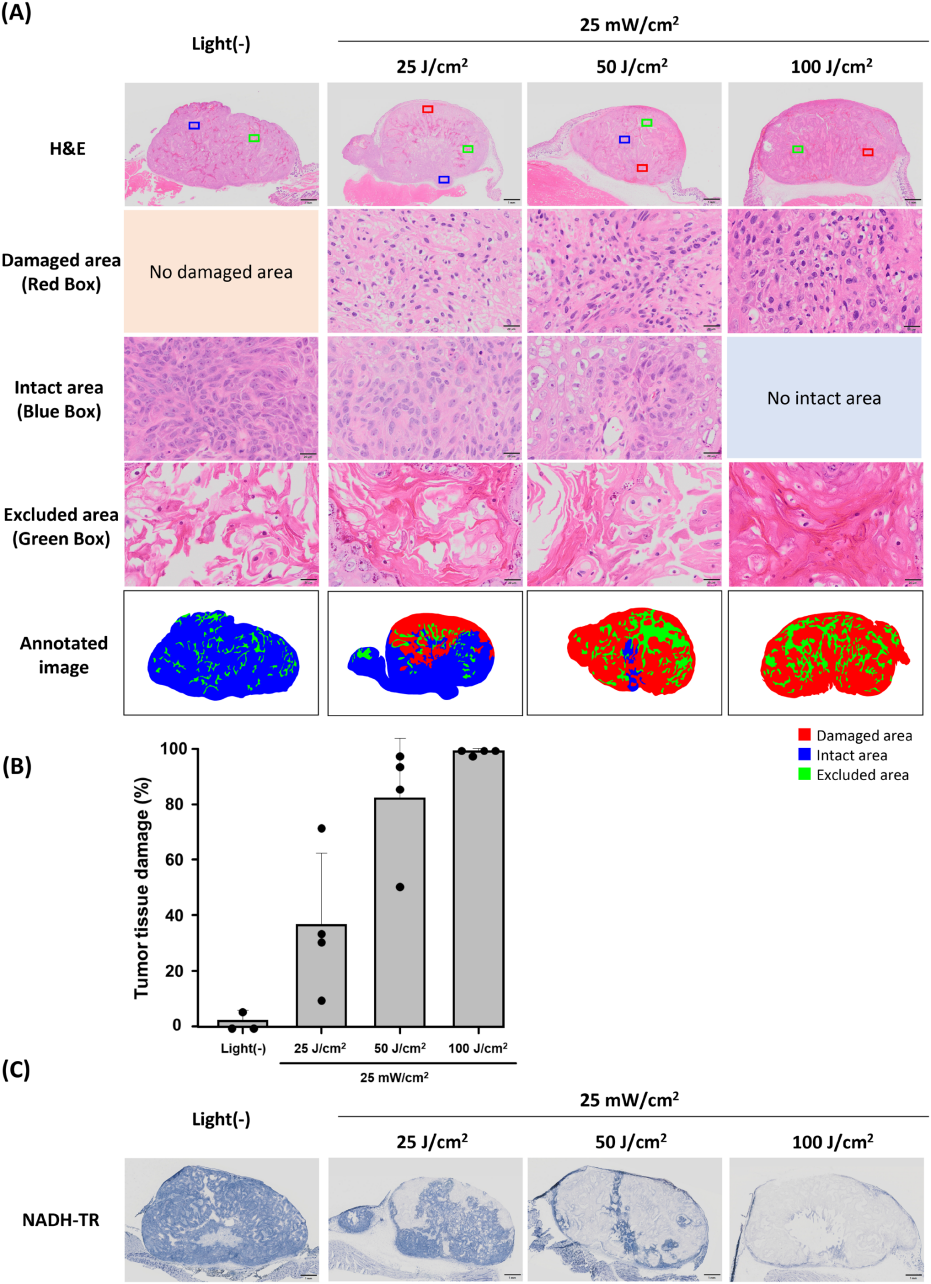
Influence of light dose on the immediate efficacy of IR700‐based PIT on A431 tumor tissue. (A) Histological images of resected A431 tumor 1 day after IR700‐based PIT. Following the defined criteria described in Materials and Methods, H&E images were annotated into three categories: Damaged area (red), intact area (blue), and area excluded from analysis (green). Boxed areas are representative examples of each category. White scale bar = 1 mm. Black scale bar = 20 μm. (B) Quantification of areas of tumor tissue damaged by IR700‐based PIT. The damaged areas of tumor tissue were scored as percentages (%) in histological images (*n* = 3–4). (C) Nicotinamide adenine dinucleotide‐tetrazolium reductase (NADH‐TR) staining images of A431 tumor sections 1 day after IR700‐based PIT. Scale bar = 1 mm.

The dose‐dependent effect of light irradiation on A431 tumor tissues was examined regarding the efficacy of IR700‐based PIT. The fluence rate was fixed at 25 mW/cm^2^ while various light irradiation doses of 25, 50, and 100 J/cm^2^ were applied. Thereafter, to evaluate tumor tissue damage, mice were euthanized 1 day after IR700‐based PIT. Tumor tissues of the Pan‐IR700‐injected group not treated with IR700‐based PIT presented a viable and highly cellular tumor, while tumor tissues of the Pan‐IR700‐injected group treated with IR700‐based PIT showed necrotic changes associated with nuclear pyknosis and fragmentation, eosinophilic cytoplasm, and micro‐hemorrhage. Histological analysis revealed an increase in the tumor tissue area showing necrotic changes with increasing light dose (Figure 3A). The percentages of necrotic area relative to the section area of tumor tissue were 9.5%–71.7% at 25 J/cm^2^, 50.9%–98.5% at 50 J/cm^2^, and 98.4%–100% at 100 J/cm^2^, respectively (Figure 3B). In the NADH staining, positively stained areas tended to match areas identified as being intact in H&E staining, while negatively stained areas tended to match necrotic areas in H&E staining (Figure 3C).

**FIGURE 4:**
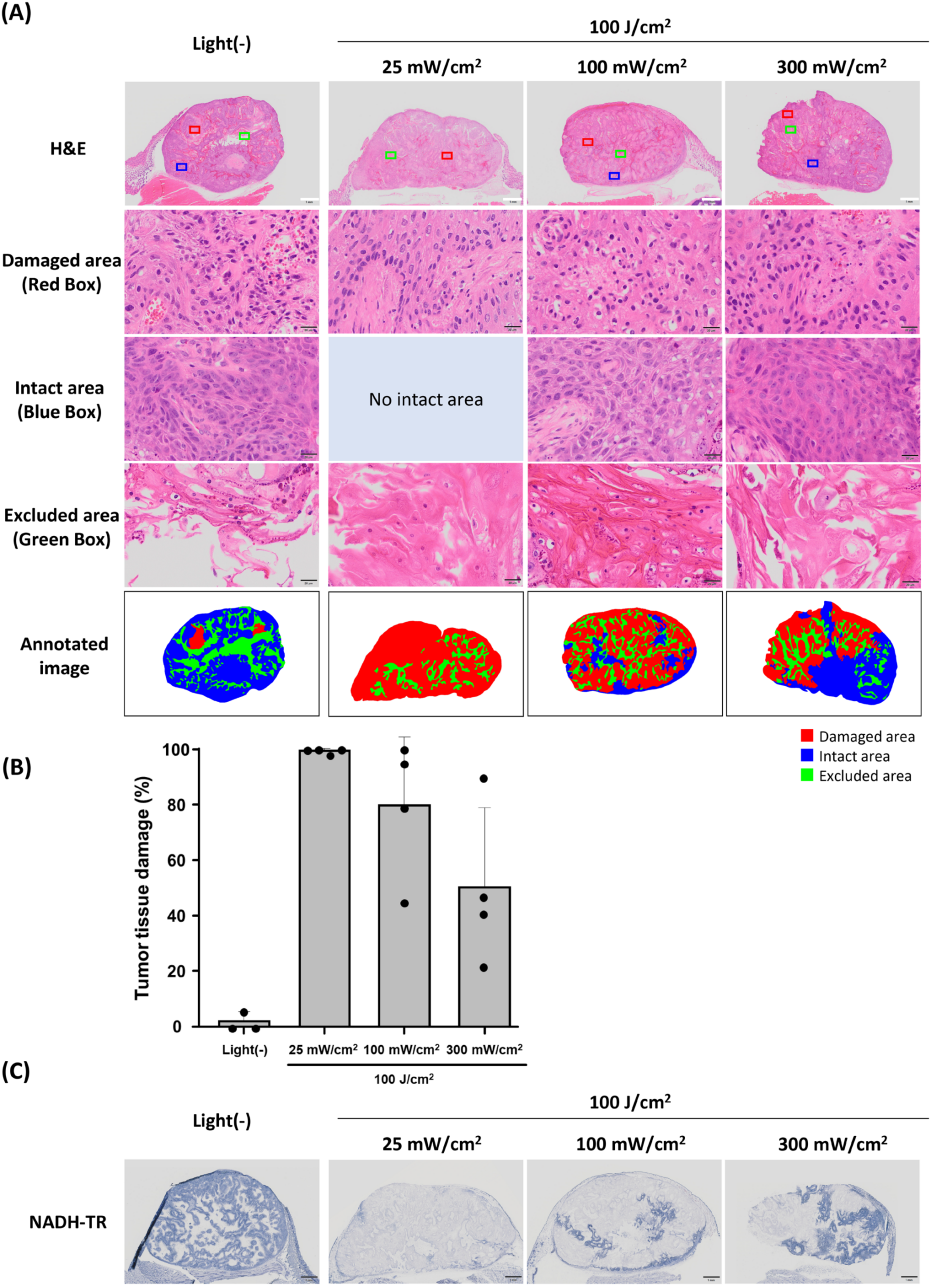
Influence of fluence rate on the immediate efficacy of IR700‐based PIT on A431 tumor tissue. (A) Histological images of resected A431 tumor 1 day after IR700‐based PIT. Following the defined criteria described in Materials and Methods, H&E images were annotated into three categories: Damaged area (red), intact area (blue), and area excluded from analysis (green). Boxed areas are representative examples of each category. White scale bar = 1 mm. Black scale bar = 20 μm. (B) Quantification of areas of tumor tissue damaged by IR700‐based PIT. The damaged areas of tumor tissue were scored as percentages (%) in histological images (*n* = 3–4). (C) NADH‐TR staining images of A431 tumor sections 1 day after IR700‐based PIT. Scale bar = 1 mm.

To investigate the impact of fluence rate on the efficacy of IR700‐based PIT, the light dose was fixed at 100 J/cm^2^ while various fluence rates of 25, 100, and 300 mW/cm^2^ were applied. Under the light irradiation conditions employed, the surface temperature of the irradiated area did not reach 50°C, and the skin surface remained unchanged visually immediately after light irradiation (Figure S4). Tumor tissues treated with IR700‐based PIT showed necrotic changes, and histological analysis revealed a decrease in tumor tissue area showing necrotic changes with increasing fluence rate (Figure 4A). The percentages of necrotic area relative to the section area of tumor tissue were 98.4%–100.0% at 25 mW/cm^2^, 45.2%–100% at 100 mW/cm^2^, and 21.8%–90.4% at 300 mW/cm^2^ (Figure 4B). In the NADH staining, positively stained areas tended to match areas identified as being intact in H&E staining, while negatively stained areas tended to match necrotic areas in H&E staining (Figure 4C).

**FIGURE 5:**
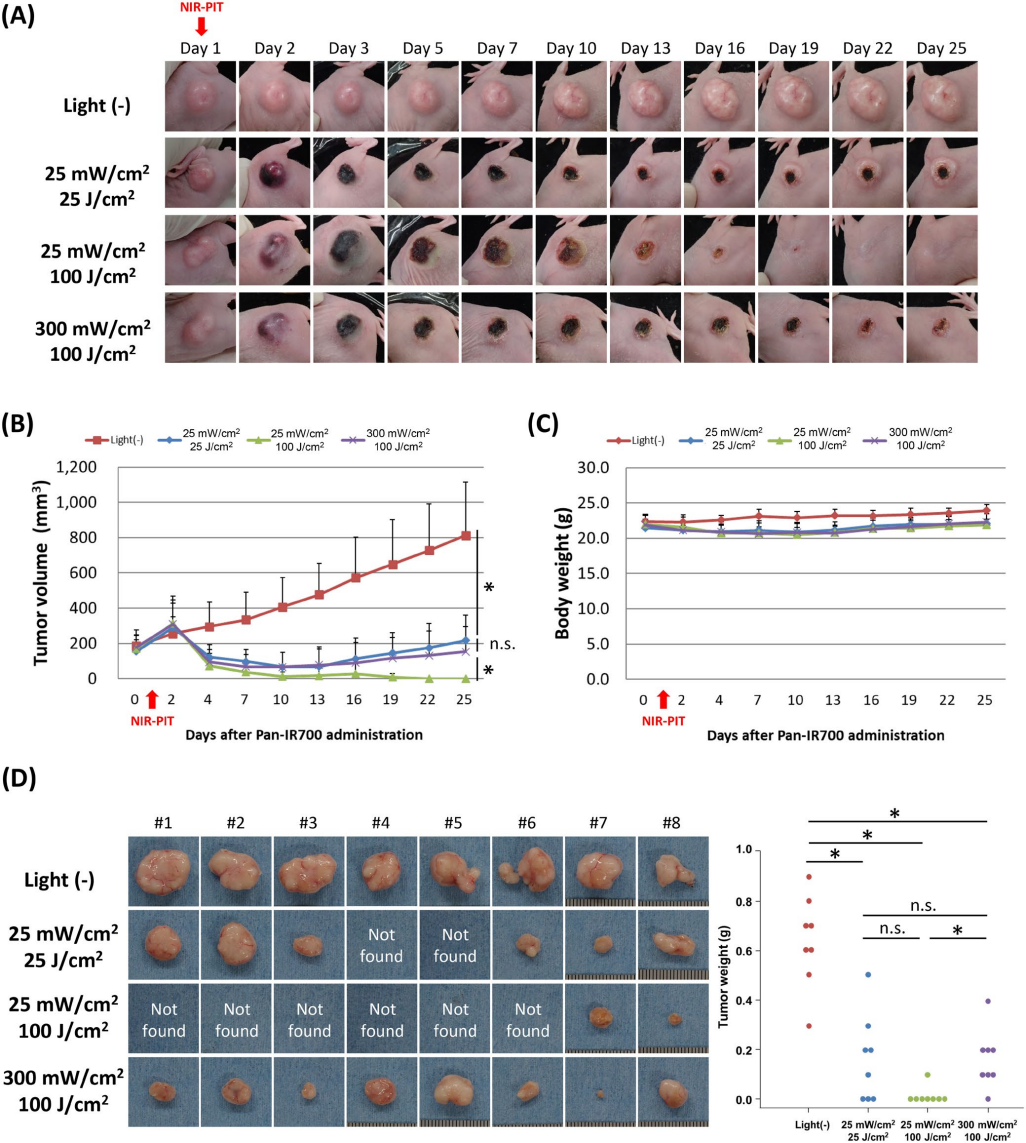
Influence of light irradiation conditions on the long‐term efficacy of IR700‐based PIT on A431 tumor tissue. (A) In vivo images of tumors after IR700‐based PIT. (B) Tumor growth inhibition in response to IR700‐based PIT (*n* = 8). In the IR700‐based PIT groups, accurate measurement of tumor diameter was difficult the day after irradiation due to edema at the irradiated site. (C) Body weight change after IR700‐based PIT. (D) Ex vivo images and tumor weights of harvested A431 tumors.

The influence of light dose and fluence rate on the efficacy of IR700‐based PIT was also analyzed in terms of the effect on tumor growth inhibition. In all light irradiation conditions examined, edema and subcutaneous hemorrhage were observed 1 day after IR700‐based PIT, resulting in the formation of crust on the skin. The crusts were then removed with wound healing (Figure 5A). In the IR700‐based PIT group, tumor growth was significantly suppressed after light irradiation. Among the conditions examined, light irradiation with the conditions of 25 mW/cm^2^ and 100 J/cm^2^ showed the highest regressive effect of implanted tumors (Figure 5B). In addition, no significant changes in body weight were observed in association with IR700‐based PIT during the follow‐up period as compared with the light (−) control group at each time point (Figure 5C). Weights of tumors harvested 24 days after IR700‐based PIT were also significantly decreased in the IR700‐based PIT group as compared with the light (−) control group. Residual tumors were found in 8 out of 8 animals with the light (−) control group; 6 out of 8 animals with the conditions of 25 mW/cm^2^ and 25 J/cm^2^ irradiation; 2 out of 8 animals with 25 mW/cm^2^ and 100 J/cm^2^ irradiation; and 8 out of 8 animals with 300 mW/cm^2^ and 100 J/cm^2^ irradiation (Figure 5D).

**FIGURE 6:**
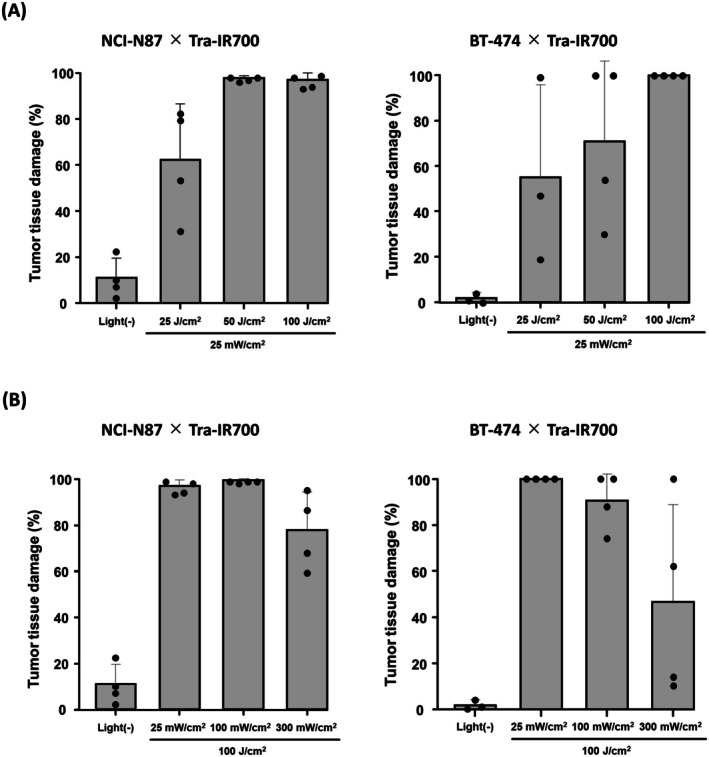
Influence of light irradiation conditions on the immediate efficacy of IR700‐based PIT in NCI‐N87 and BT‐474 tumor tissue. Evaluation of IR700‐based PIT efficacy in NCI‐N87 and BT‐474 tumor tissue. The tissue damage was scored as percentages (%) in histological images (*n* = 3–4). The examined parameters of the light irradiation conditions were (A) light dose and (B) fluence rate.

The influence of light dose and fluence rate on the efficacy of IR700‐based PIT was examined using two HER2‐targeted IR700‐based PIT animal models. NCI‐N87 cells treated with Tra‐IR700 showed Tra‐IR700 fluorescence (Figure S5), and NCI‐N87 tumor‐bearing mice administered Tra‐IR700 showed the fluorescence of Tra‐IR700 in the tumor tissues (Figure S6). Histological analysis revealed increases in the areas of BT‐474 and NCI‐N87 tumor tissues showing necrotic changes with increasing light dose (Figures 6A, S7), while these areas decreased with increasing fluence rate (Figures 6B, S8), in accordance with the findings for A431 tumor tissue treated with Pan‐IR700.

## 4 Discussion

In the current study, the fluorescence of synthesized Pan‐IR700 was found on the cell surface membrane, indicating that Pan‐IR700 can bind specifically to the EGFR protein on the plasma membrane of cancer cells, consistent with the findings in previous studies using Pan‐IR700 in other cell lines [17, 25, 26]. Hoechst/PI double‐staining and LDH assay revealed that the efficacy of IR700‐based PIT in vitro increases in a light dose‐dependent manner, consistent with previous reports [21, 27, 28]. However, the efficacy of IR700‐based PIT did not differ significantly in a manner depending on the fluence rate. These results suggest that fluence rate is not related to the efficacy of IR700‐based PIT in vitro. In addition, the cellular distribution of Pan‐IR700 changed over time after incubation. Pan‐IR700 was initially bound to EGFR on the cell surface membrane and then translocated to the lysosome by internalization. In the present study, we demonstrated that the internalized Pan‐IR700 into lysosomes also contributed to cell death in IR700‐based PIT. However, the cytotoxic effect of Pan‐IR700 localized within the lysosomes at the highest light dose was lower than that caused by cell surface membrane damage. These results suggest that IR700‐based PIT targeting the cell surface membrane is more effective than that targeting lysosomes.

In vivo imaging revealed that intravenously administered Pan‐IR700 accumulated in grafted A431 subcutaneous tumors 1 day after administration, consistent with previous reports [4, 11, 29]. At this time point, the fluorescence signal of Pan‐IR700 visually matched well to the immunofluorescent signal of EGFR showing the A431 tumor cell membrane. In the colocalization analysis of Pan‐IR700 and EGFR signals, A431 tumors in animals with Pan‐IR700 injection showed a high Pearson's R value, indicating a linear correlation between fluorescence signals of Pan‐IR700 and EGFR. With regard to Mander's tM1 parameter, an indicator of the colocalization coefficient between the two images, we found an increase in this value in A431 tumor sections in animals with Pan‐IR700 injection compared to those without Pan‐IR700 injection. These results suggest that the administered Pan‐IR700 bound to EGFR molecules on the surface of the A431 tumor cells. In the present study, the necrotic area relative to the section area of tumor tissue 1 day after IR700‐based PIT increased in a light dose‐dependent manner. This suggests that the efficacy of IR700‐based PIT depends on the light dose, in agreement with previous reports [17, 19, 20, 21]. However, we also found that variability in the necrotic area of tumor tissue increased when the light dose was decreased. In accordance with this variability, there was a two‐fold difference in the IR700 fluorescence signal in the tumor site at 1 day after administration, although the dose of mAb‐IR700 was constant. The variability in the IR700 fluorescence signal may reflect the uneven accumulation of administered mAb‐IR700 in tumor tissue. While it has been reported that the cytotoxicity was enhanced in a mAb‐IR700 concentration‐dependent manner in a light dose of 2 J/cm^2^ in vitro [12], we previously found that the cytotoxicity of mAb‐IR700 concentration dependency was not observed at a high light dose of 10 J/cm^2^ [30]. Therefore, the in vivo anti‐tumor effect of IR700‐based PIT may be influenced by the amount of mAb‐IR700 delivered to the tumor under low light dose conditions resulting in the variability in the necrosis effect. Besides, the necrotic area relative to the section area of tumor tissue 1 day after IR700‐based PIT decreased with increasing fluence rate. This suggests that light irradiation at low fluence rates enhances the efficacy of IR700‐based PIT in vivo. Furthermore, tumor growth inhibition at 24 days after light irradiation was enhanced in a light dose‐dependent manner and decreased with increasing fluence rate, suggesting that the immediate efficacy as revealed by histological evaluation reflects the final anti‐tumor effect. Under the light irradiation conditions employed in this study, the surface temperature of an irradiated area did not reach 50°C, at which skin burns were induced in a previous study [31], regardless of Pan‐IR700 administration. Therefore, it is considered that skin burn‐induced tissue damage was unlikely to occur to influence the efficacy of IR700‐based PIT under the light irradiation conditions applied in this study.

For the IR700‐based PIT model (i.e., the pairing of target cells with mAb‐IR700) in our study, the efficacy of IR700‐based PIT was assessed after verifying that the synthesized mAb‐IR700 binds to the target cells and accumulates in the tumor tissue upon administration, as designed [23]. The IR700‐based PIT efficacy increased in a light dose‐dependent manner and was suppressed by increasing fluence rate in all three IR700‐based PIT animal models examined. These results suggest that the influence of light irradiation conditions on IR700‐based PIT is independent of the type of tumor cells or mAb‐IR700, with a suitable combination of target molecule and mAb‐IR700.

In the present study, mAb‐IR700 administered in vivo was bound to tumor cells; however, their distribution was heterogeneously observed in the tumor tissues. Nevertheless, tumor tissues were entirely damaged after 1 day of light irradiation at 25 mW/cm^2^ and 100 J/cm^2^, resulting in a high anti‐tumor effect. In addition, transient edema and subcutaneous hemorrhage were observed in irradiated areas after IR700‐based PIT, and these changes were evidently observed in association with the anti‐tumor effect. These results suggest that in vivo IR700‐based PIT induces changes in the tumor microenvironment as well as direct damage to tumor cells, and light irradiation conditions may influence the effect on both tumor cells and the tumor environment. Previous studies have suggested that mAb‐IR700 unbound to tumor cell surfaces produces cytotoxic effects on the vascular endothelial cells in irradiated areas [32], causing indirect anti‐tumor effects by vascular occlusion [33]. Meanwhile, in photodynamic therapy, computational science analysis using a mathematical model revealed that exceeding oxygen consumption for the formation of reactive oxygen species rather than oxygen supply from blood during light irradiation at a high fluence rate diminishes the anti‐tumor effect caused by photooxidation [34]. Since it is known that mAb‐IR700 produces reactive oxygen species [33], the efficacy of IR700‐based PIT might be influenced by fluence rate in the same manner as photodynamic therapy. However, we consider that IR700‐based PIT is effective as a molecular targeted therapy because a previous study demonstrated that target molecule‐expressing tumor cells were selectively disrupted in the presence of target molecule‐negative tumor cells in a mixed tumor model [35].

With regard to the effect of fluence rate on the IR700‐based PIT efficacy, we found inconsistency between in vitro and in vivo models in the present study. A previous study has shown that the efficacy of in vitro and in vivo IR700‐based PIT increased with decreasing fluence rates [5]. However, the present study showed that the efficacy of in vitro IR700‐based PIT was not affected by fluence rate in the examination, including the similar conditions applied in that reported study. These findings suggest that the effect of fluence rate on the efficacy of in vivo IR700‐based PIT might be derived from tumor environments other than tumor cells themselves. In the tissue environment, tumor cells interact with various kinds of tissue components, such as the cancer stroma, including blood supply and the immune system, to control complex biological functions; however, the in vitro environment cannot reproduce the in vivo tissue microenvironments. Therefore, as aforementioned, it is reasonable to consider that the effects of IR700‐based PIT on the tumor microenvironment are affected by fluence rate that may be responsible for the observed inconsistency between in vitro and in vivo studies.

Several issues from this study remain unresolved. First, the efficacy of IR700‐based PIT was examined only in tumor tissue in the present study. Although IR700‐based PIT is a highly cell‐selective platform, side effects such as treatment‐induced edema were observed in this study and clinical practice [36]. Therefore, the optimal light irradiation conditions in IR700‐based PIT need to be determined with an adequate understanding of the effects on tumor tissue as well as on normal tissue. To do this, it is necessary to address the influence of light exposure conditions on the efficacy of IR700‐based PIT for normal tissues, as examined in tumor tissues. Second, the animal models of IR700‐based PIT used in this study were created using immunodeficient mice. When considering the therapeutic effect of IR700‐based PIT, which has been reported to activate cancer immunity by immunogenic cell death [37, 38, 39], the response by immune cells should be taken into account. Also, because immune cell kinetics can change the constitution of the tumor microenvironment, the immunodeficient model used in this study may differ in the effect of light irradiation conditions on the efficacy of IR700‐based PIT from the immunocompetent model. Therefore, for accurate assessment of the influence of light irradiation conditions on the therapeutic effect of IR700‐based PIT, it is desirable to use an immunocompetent model. Third, we could not examine the parameters involved in mAb‐IR700 in this study. IR700‐based PIT is performed in combination with the infusion of mAb‐IR700 and light irradiation; thus, parameters related to mAb‐IR700 would also influence the therapeutic effects. However, since this study focused on the light irradiation conditions, we had to keep the mAb‐IR700‐related parameters constant.

In the present study, light irradiation at lower fluence rates showed better IR700‐based PIT efficacy. In clinical practice, superficial irradiation is performed at a fluence rate of 150 mW/cm^2^, which does not cause thermal damage [40]. Thus, light irradiation at fluence rates lower than those currently applied may improve the therapeutic outcome of IR700‐based PIT in clinical practice. Endoscopic light irradiation is a good approach when considering IR700‐based PIT for the gastrointestinal tract, lungs, or body cavities such as the bladder, abdominal cavity, or pleural cavity. Actually, the anti‐tumor effect of IR700‐based PIT can be achieved by light irradiation with a fiber optic diffuser through the forceps channel of an endoscope inserted into the oral or abdominal cavity of mice [41, 42, 43]. This suggests that IR700‐based PIT using a conventional endoscopic light source also achieves a therapeutic outcome. However, light irradiation at low fluence rates requires a prolonged duration of treatment. Endoscopic manipulation produces immense shakes due to the operator's camera shake or the patient's peristaltic motion [44]. As the duration of treatment prolongs, it becomes difficult to hold the irradiation area at an aimed position, which may cause variability in the irradiation dose. Therefore, it is possible that light irradiation at the low fluence rate does not reach the dose required for treatment, resulting in insufficient treatment areas in clinical practice. Recently, a wireless light system emitting light at 680–700 nm at a fluence rate of 1.3 mW/cm^2^ was newly developed as a stable light source for internal or interstitial light exposure [45]. For light irradiation of tumors at low fluence rates over a long period of time, implantation of a wirelessly powered LED via an endoscope channel is considered to be useful as a solution to cover insufficient treatment. However, it should be noted that the light irradiation conditions showing apparent anti‐tumor effects may be related to those inducing intense edema at the irradiated area. Thus, control of adverse events might be necessary in clinical practice.

In conclusion, our results demonstrated that the efficacy of IR700‐based PIT in cultured cells increased in a light dose‐dependent manner and was not influenced by fluence rate, while its efficacy in three IR700‐based PIT animal models increased in a light dose‐dependent manner and was suppressed by increasing fluence rate. Therefore, to optimize the therapeutic efficacy of IR700‐based PIT for tumor tissue, light irradiation at lower fluence rates could be recommended.

## Author Contributions

**Susumu Yamashita:** conceptualization (equal), data curation (equal), formal analysis (equal), investigation (equal), methodology (equal), visualization (equal), writing – original draft (equal), writing – review and editing (equal). **Miho Kojima:** conceptualization (equal), data curation (equal), methodology (equal), writing – review and editing (equal). **Nobuhiko Onda:** conceptualization (equal), data curation (equal), methodology (equal), writing – review and editing (equal). **Makoto Shibutani:** conceptualization (equal), formal analysis (equal), supervision (equal), validation (equal), writing – review and editing (equal).

## Ethics Statement

All animal experiments in the current study were performed in accordance with the Guidelines for Proper Conduct of Animal Experiments (Science Council of Japan, 1 June, 2006) and with the protocol approved by the Animal Care and Use Committee of Tokyo University of Agriculture and Technology (approval no.: 30–97, 30–136, R05‐208, R06‐78). All efforts were made to minimize animal suffering.

## Consent

The authors have nothing to report.

## Conflicts of Interest

Susumu Yamashita, Miho Kojima, and Nobuhiko Onda are employees of Olympus Medical Systems Corp., which supported this work. The other authors have no potential conflicts of interest to disclose.

## Supporting information


**Table S1:** List of cell lines used in this study.
**Table S2:** Antibodies used in this study.
**Table S3:** List of fluorescent organelle markers used in this study.
**Table S4:** In vivo IR700‐based PIT group with light irradiation conditions and number of samples.
**Figure S1:** Influence of light irradiation conditions on efficacy of IRDye700DX‐based photoimmunotherapy (IR700‐based PIT) in vitro.
**Figure S2:** Cellular localization of Pan‐IR700 and cytotoxicity of IR700‐based PIT in A431 cells.
**Figure S3:** EGFR immunoreactivity in the xenografted A431 tumor tissue.
**Figure S4:** Fluorescence imaging and colocalization analysis in A431 tumors.
**Figure S5:** Responses of NCI‐N87 cells to synthesized trasutuzumab‐IRDye700DX (Tra‐IR700).
**Figure S6:** Fluorescence imaging of administered Tra‐IR700 in NCI‐N87 tumors.
**Figure S7:** Influence of light dose on the efficacy of IR700‐based PIT in NCI‐N87 and BT‐474 tumor tissue.
**Figure S8:** Influence of fluence rate on the efficacy of IR700‐based PIT in NCI‐N87 and BT‐474 tumor tissue.

## Data Availability

The data that support the findings of this study are available from the corresponding author uponreasonable request.
