# Supplementary material for: Effect of Fluence Rate on Tumor Tissue Damage in IRDye700DX‐Based Photoimmunotherapy
Source: Cancer Med. 2025 Aug 19;14(16):e71143. doi: 10.1002/cam4.71143 (PMC12364714; doi:10.1002/cam4.71143)
Supplement: Supplementary file 1 — Table S1: List of cell lines used in this study. Table S2: Antibodies used in this study. Table S3: List of fluorescent organelle markers used in this study. Table S4: In vivo IR700‐based PIT group with light irradiation conditions and number of samples. Figure S1: Influence of light irradiation conditions on efficacy of IRDye700DX‐based photoimmunotherapy (IR700‐based PIT) in vitro. Figure S2: Cellular localization of Pan‐IR700 and cytotoxicity of IR700‐based PIT in A431 cells. Figure S3: EGFR immunoreactivity in the xenografted A431 tumor tissue. Figure S4: Fluorescence imaging and colocalization analysis in A431 tumors. Figure S5: Responses of NCI‐N87 cells to synthesized trasutuzumab‐IRDye700DX (Tra‐IR700). Figure S6: Fluorescence imaging of administered Tra‐IR700 in NCI‐N87 tumors. Figure S7: Influence of light dose on the efficacy of IR700‐based PIT in NCI‐N87 and BT‐474 tumor tissue. Figure S8: Influence of fluence rate on the efficacy of IR700‐based PIT in NCI‐N87 and BT‐474 tumor tissue. [file CAM4-14-e71143-s001.docx]

**Supporting Information**

**Article title:** Effect of fluence rate on tumor tissue damage in IRDye700DX-based photoimmunotherapy

**Authors:** Susumu Yamashita, Miho Kojima, Nobuhiko Onda, and Makoto Shibutani

**The Supporting Information includes:**

**Supporting Information Figure S1–9**

**Supporting Information Tables S1-4**

**Corresponding authors: Makoto Shibutani (mshibuta@cc.tuat.ac.jp)**

**
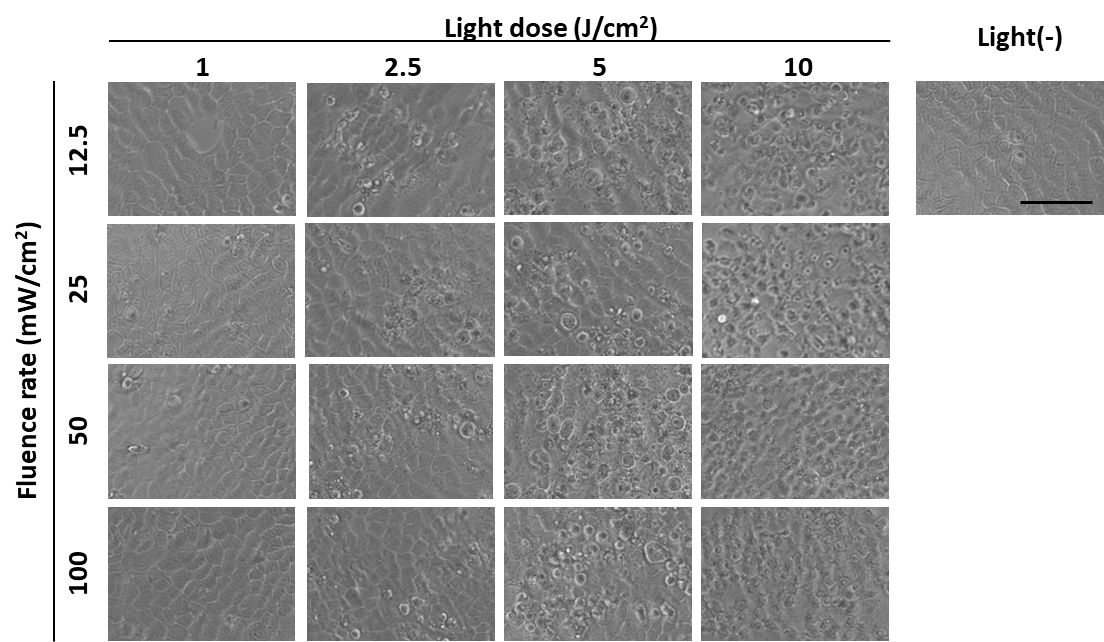
**

**Figure S1. Influence of light irradiation conditions on efficacy of IRDye700DX-based photoimmunotherapy (IR700-based PIT) in vitro.** Phase contrast images 1 day after IR700-based PIT in A431 cells exposed to various light irradiation conditions. Scale bar = 100 μm.

**
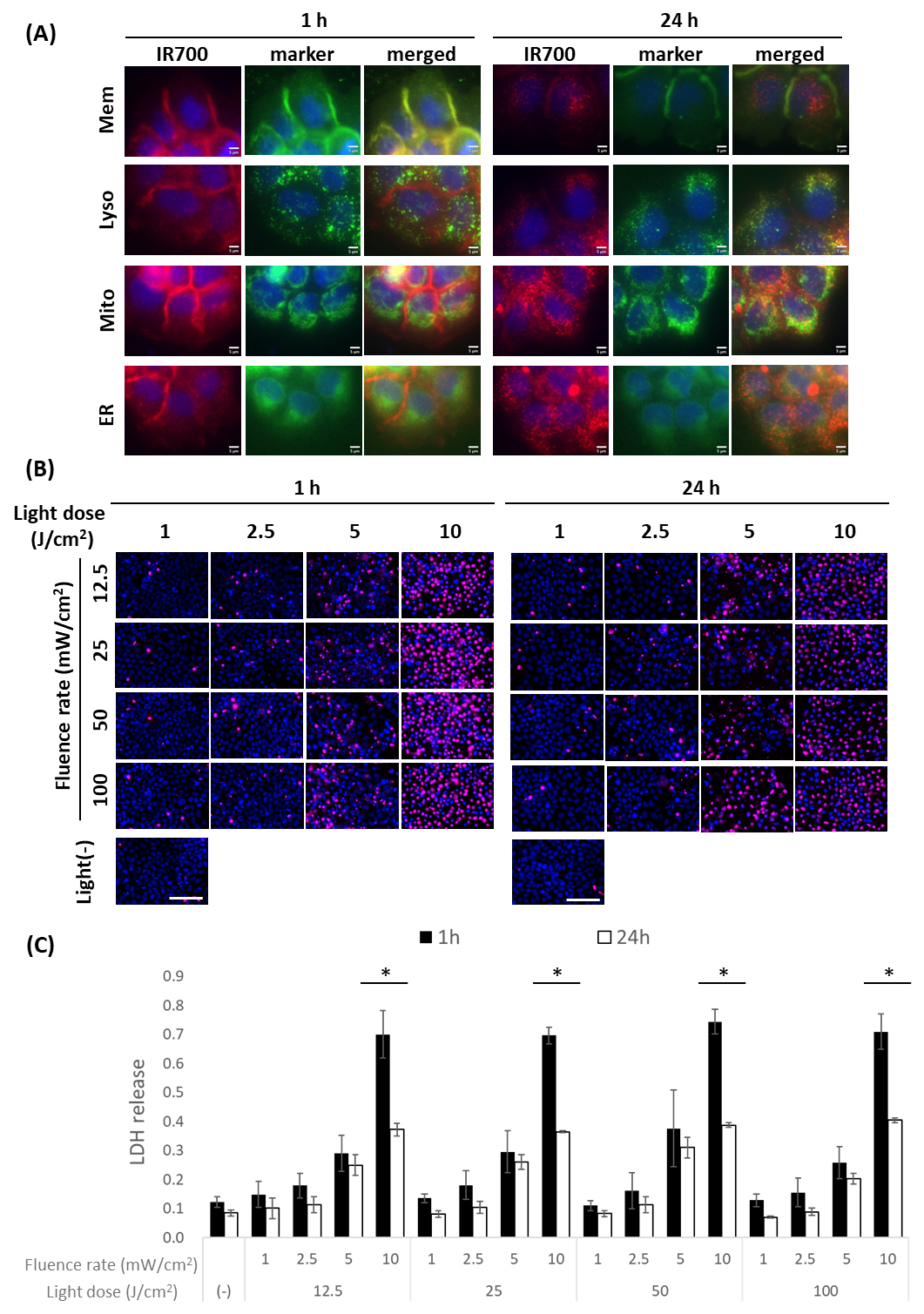
**

**Figure S2. Influence of light irradiation conditions on efficacy of IR700-based PIT in vitro.** (A) Cellular localization of panitumumab-IRDye700DX (Pan-IR700). After incubation with Pan-IR700 (red) for 1 or 24 h, Each organelle was stained with the respective probe (green). Mem: Cell membrane; Lyso: lysosome; Mito: mitochondria; ER: endoplasmic reticulum. Nuclei were stained with Hoechst 33342 (blue). Scale bar = 5 μm. (B) Hoechst 33342 (blue) and propidium iodide (red) double-stained images 1 day after IR700-based PIT in A431 cells exposed to various conditions. Scale bar = 100 μm. (C) Lactate dehydrogenase (LDH) assay 1 day after IR700-based PIT in A431 cells exposed to various light irradiation conditions. Data are presented as mean ± SD (n = 3, Tukey’s test; * statistically significant difference from control group at p < 0.05).

**
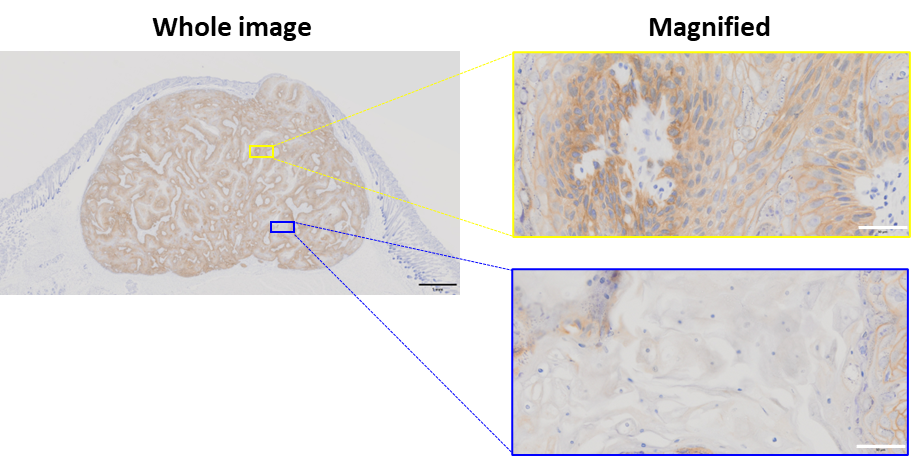
**

**Figure S3. EGFR immunoreactivity in the xenografted A431 tumor tissue.** EGFR immunoreactivity in A431 tumors was high in the basal cell layer (yellow box) and low in keratinocytes (blue box). Sections were made from formalin-fixed, paraffin-embedded tumor samples. Deparaffinized sections were subjected to antigen retrieval treatment by microwaving in SignalStain EDTA unmasking solution (Cell Signaling Technology, Beverly, MA, USA) and quenching endogenous peroxidase by incubating in 0.3% hydrogen peroxide solution in absolute methanol. Immunohistochemical staining was performed using primary antibody against EGFR (Cell Signaling Technology) and Peroxidase-conjugated secondary antibody (Histofine Simple Stain MAX PO (R); Nichirei, Tokyo, Japan). Color development was performed with 3,3’-diaminobenzidine tetrahydrochloride/H_2_O_2_ in Tris-buffered saline (pH 7.6) as the chromogen. Nuclear counterstaining was conducted with hematoxylin and cover-slipped for microscopic examination. Black scale bar = 1 mm, White scale bar = 50 μm

**
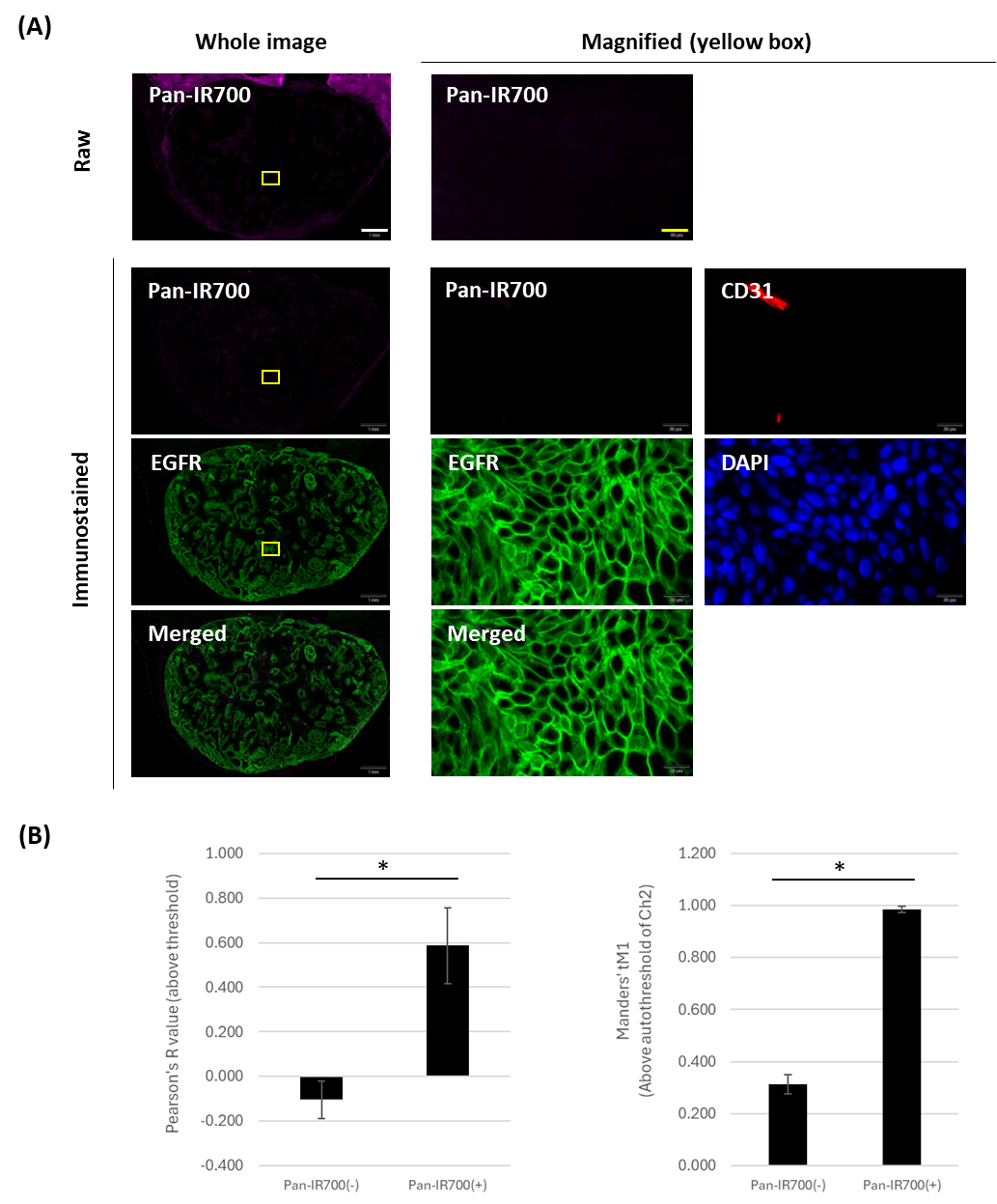
**

**Figure S4. Fluorescence imaging and colocalization analysis in A431 tumors.** (A) Fluorescence images of tissue section for A431 tumors without Pan-IR700 injection. The top panel shows fluorescence image of Pan-IR700 (magenta) in unfixed frozen sections. The panels from the second row onwards are fluorescent immunostaining images of the frozen sections. The colors of each image correspond to the stained molecules as follows: panitumumab (magenta), epidermal growth factor receptor (green), cluster of differentiation 31 (red), and DAPI (blue). White scale bar = 1 mm. Yellow scale bar = 20 μm. (B) Image analysis of the colocalization correlation parameters. To evaluate colocalization between Pan-IR700 and epidermal growth factor receptor, we employed Pearson’s R value and Mander's tM1 parameter. Colocalization analysis was performed using the coloc2 plug-in of ImageJ by randomly selecting five fields of view from the whole image of EGFR-fluorescent immunostaining. Data are presented as means ± SD (n = 3; Student's *t*-test; *p < 0.05).


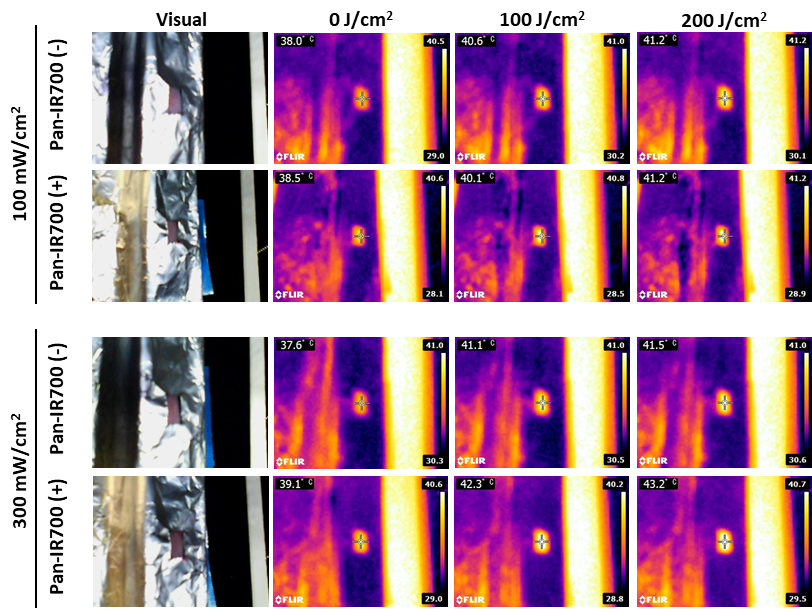


**Figure S5. Preliminary examination of surface temperature changes during light irradiation at fluence rates of 100 mW/cm^2^ or 300 mW/cm^2^.** Mice injected with Pan-IR700 one day before or without Pan-IR700 injection were anesthetized with isoflurane and placed on a heat pad for 30 min to exclude hypothermic effect by anesthesia. While the mice were exposed to light at each fluence rate, the surface temperature of the irradiated area was measured with a spot meter in a FLIR E4 thermal imaging camera (FLIR Systems, Inc., Wilsonville, OR, USA).

**
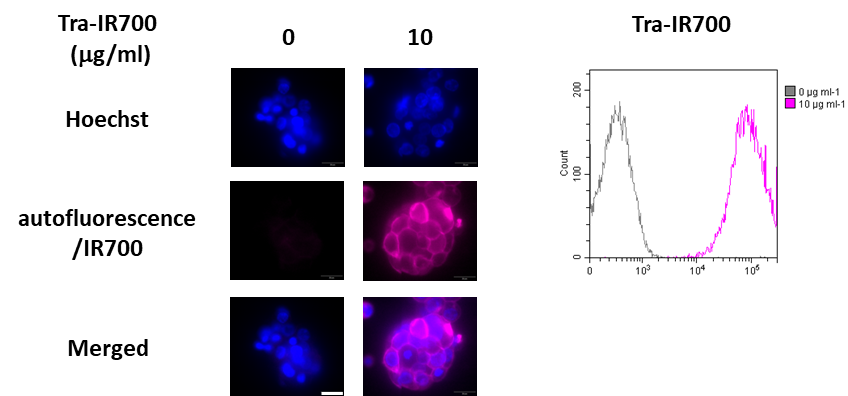
Figure S6. Responses of NCI-N87 cells to synthesized trasutuzumab-IRDye700DX (Tra-IR700).** Fluorescence images and flow cytometry analysis of NCI-N87 cells incubated with or without Tra-IR700. Scale bar = 20 μm.


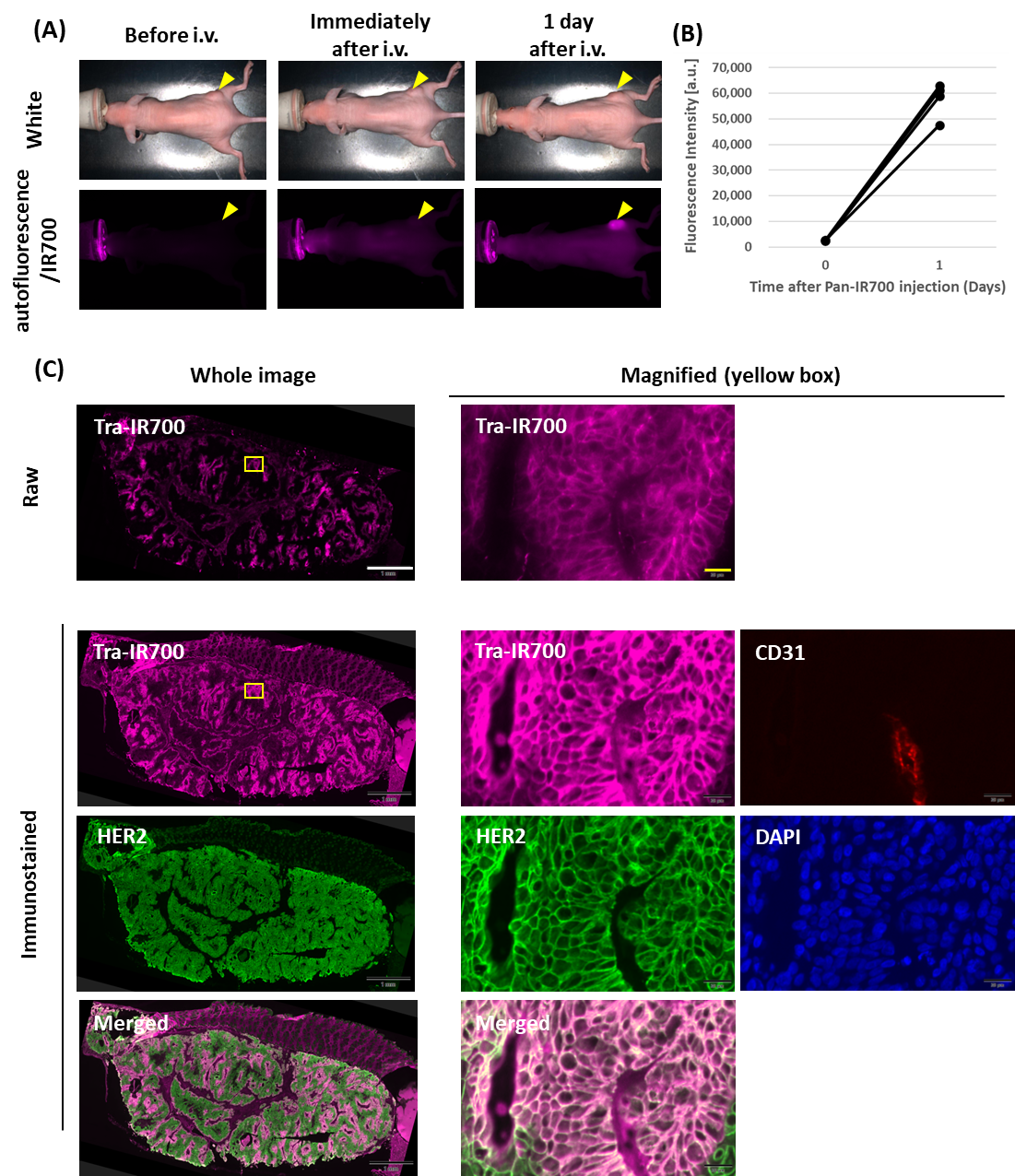


**Figure S7. Fluorescence imaging of administered Tra-IR700 in NCI-N87 tumors.** (A) Time-course imaging of Tra-IR700 fluorescence in NCI-N87 tumor-bearing mice (right dorsum as shown by yellow arrow). Before i.v. image indicates the autofluorescence signal. (B) Changes in the fluorescence signal intensity by the administration of Tra-IR700 (n = 4). (C) Tissue and cellular distribution of Tra-IR700 in NCI-N87 tumors 1 day after injection. The top panel shows fluorescence image of administered Tra-IR700 (magenta) in unfixed frozen sections. The panels from the second row onwards are fluorescent immunostaining images of the frozen sections. The colors of each image correspond to the stained molecules as follows: trastuzumab (magenta), human epidermal growth factor receptor 2 (green), cluster of differentiation 31 (red), and DAPI (blue). The merged image of trastuzumab and human epidermal growth factor receptor 2 was generated to show merged signals as white in color. White scale bar = 1 mm. Yellow scale bar = 20 μm.

**
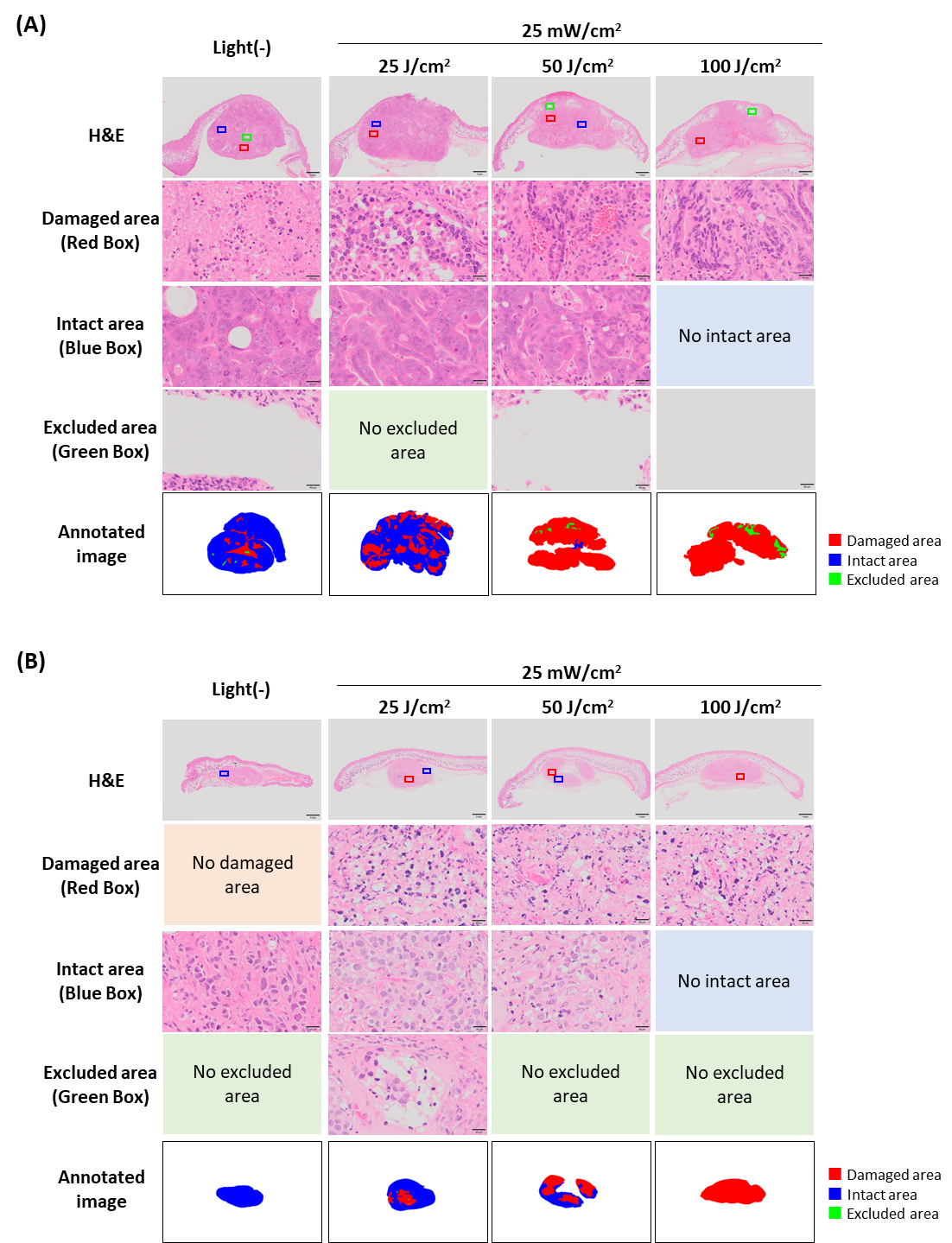
**

**Figure S8. Influence of light dose on the efficacy of IR700-based PIT in NCI-N87 and BT-474 tumor tissue.** Histological images of resected tumor 1 day after IR700-based PIT. (A) NCI-N87 tumor, (B) BT-474 tumor. Following the defined criteria described in Materials and Methods, H&E images were annotated into three categories: damaged area (red), intact area (blue), and area excluded from analysis (green). Boxed areas are representative examples of each category. White scale bar = 1 mm. Black scale bar = 20 μm.


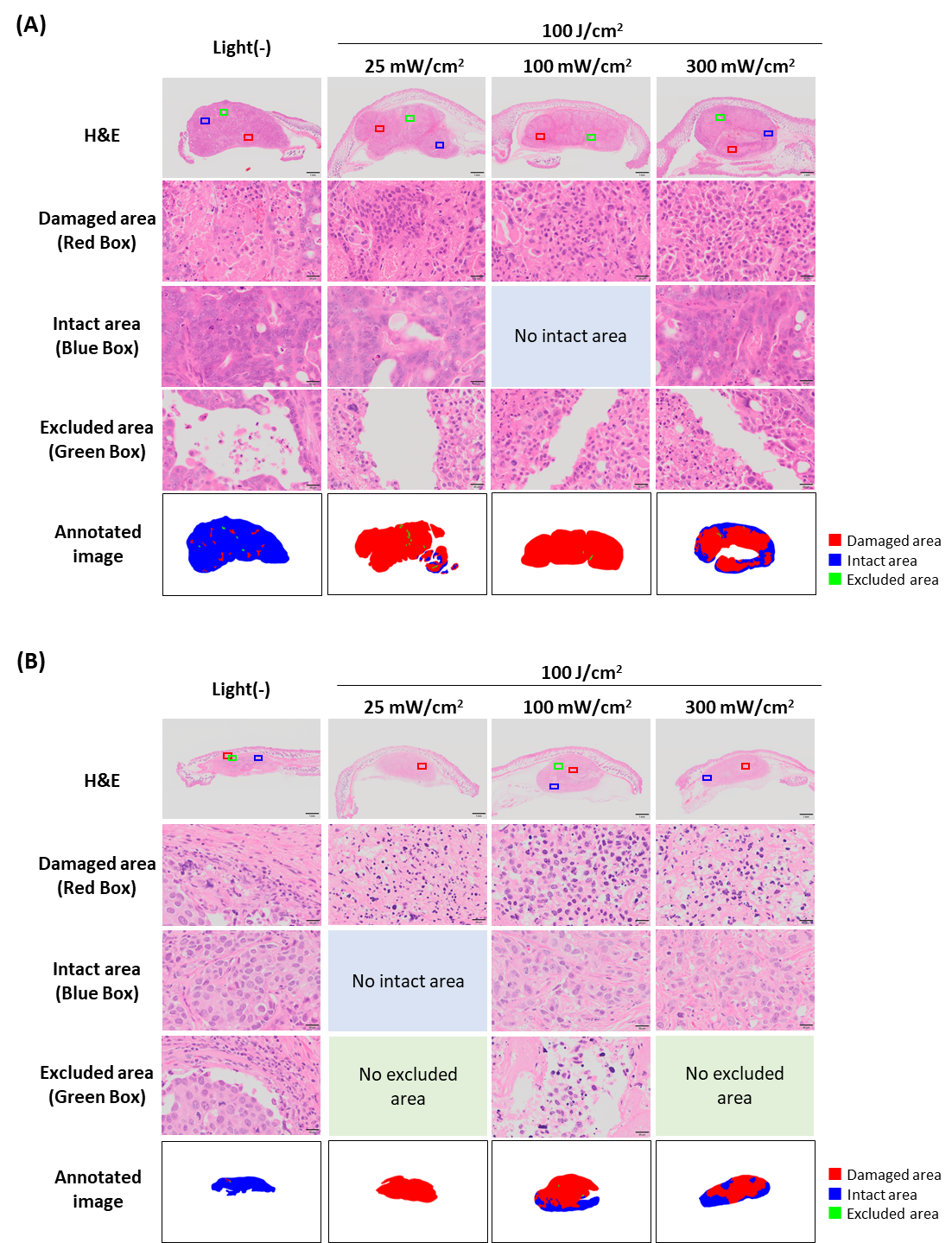


**Figure S9. Influence of fluence rate on the efficacy of IR700-based PIT in NCI-N87 and BT-474 tumor tissue.** Histological images of resected tumor 1 day after IR700-based PIT. (A) NCI-N87 tumor, (B) BT-474 tumor. Following the defined criteria described in Materials and Methods, H&E images were annotated into three categories: damaged area (red), intact area (blue), and area excluded from analysis (green). Boxed areas are representative examples of each category. White scale bar = 1 mm. Black scale bar = 20 μm.

**Table S1. List of cell lines used in this study.**

| Cell line | Origin | Culture medium* |
| --- | --- | --- |
| A431 | European Collection of Authenticated Cell Cultures through DS Pharma Biomedical (Osaka, Japan) | DMEM (10569010; Thermo Fisher Scientific) |
| NCI-N87 | American Type Culture Collection through Summit Pharmaceuticals International Corporation (Tokyo, Japan) | RPMI-1640 medium (61870036; Thermo Fisher Scientific) |
| BT-474 | American Type Culture Collection through Summit Pharmaceuticals International Corporation | DMEM |

*All media were supplemented with 10% fetal bovine serum (26140079; Thermo Fisher Scientific), 100 U penicillin/ml, and 0.1 mg streptomycin/ml (15140122; Thermo Fisher Scientific), at 37°C in a 5% CO2 humidified atmosphere.

**Table S2. Antibodies used in this study.**

|  | Abbreviated name | Manufacturer (City, State, Country) | Product # | Host species | Dilution |
| --- | --- | --- | --- | --- | --- |
| Antigen |  |  |  |  |  |
| epidermal growth factor receptor | EGFR | Cell Signaling Technology (Beverly, MA, USA) | 4267 | Rabbit | 1:100 |
| human epidermal growth factor receptor 2 | HER2 | Cell Signaling Technology | 2165 | Rabbit | 1:100 |
| cluster of differentiation 31 | CD31 | BD harmingen (San Diego, CA, USA) | 550274 | Rat | 1:100 |
| Secondary antibody |  |  |  |  |  |
| Alexa Fluor 488 anti-Rat IgG (H+L) | － | Thermo Fisher Scientific (Walthan, MA, USA) | A21208 | Donkey | 1:500 |
| Alexa Fluor 568 anti-Rabbit IgG (H+L) | － | Thermo Fisher Scientific | A10042 | Donkey | 1:500 |
| Alexa Fluor 647 anti-Human IgG (H+L) | － | Thermo Fisher Scientific | A21445 | Goat | 1:500 |

**Table S3. List of fluorescent organelle markers used in this study.**

| Organelle | Probes for organelle * | Manufacturer (City, State, Country) | Product # | Concentration |
| --- | --- | --- | --- | --- |
| Membrane | MemGlow™ 488 | Cytoskeleton, Inc. (Denver, CO, USA) | MG02-02 | 100 nM |
| Lysosome | LysoTracker® Green DND-26 | Thermo Fisher Scientific (Walthan, MA, USA) | L7526 | 1 μM |
| Mitochondria | MitoTracker® Green FM | Thermo Fisher Scientific | M7514 | 200 nM |
| Endoplasmic reticulum | ER-TrackerTM Green  (BODIPY® FL Glibenclamide) | Thermo Fisher Scientific | E34251 | 1 μM |

*Live-staining procedures were carried out after Pan-IR700 incubation and according to the manufacturer’s instructions.

**Table S4. In vivo IR700-based PIT group with light irradiation condition and number of samples.**

| BT-474 | histological evaluation | Sample number | 3 | 3 | 4 | 4 | 4 | 4 |
| --- | --- | --- | --- | --- | --- | --- | --- | --- |
|  |  | Images used for | Figure S5, S6 | Figure S5 | Figure S5 | Figure S5, S6 | Figure S5 | Figure S5 |
| NCI-N87 | histological evaluation | Sample number | 4 | 4 | 4 | 4 | 4 | 4 |
|  |  | Images used for | Figure S5, S6 | Figure S5 | Figure S5 | Figure S5, S6 | Figure S5 | Figure S5 |
| A431 | histological evaluation | Sample number | 3 | 4 | 4 | 4 | 4 | 4 |
|  |  | Images used for | Figure 3, 4 | Figure 3 | Figure 3 | Figure 3, 4 | Figure 4 | Figure 4 |
|  | tumor growth inhibition | Sample number | 8 | 8 | － | 8 | － | 8 |
|  |  | Images used for | Figure 5 | Figure 5 | － | Figure 5 | － | Figure 5 |
| Irradiation  time [s] | | | － | 1000 | 2000 | 4000 | 1000 | 667 |
| Light dose [J/cm2] | | | － | 25 | 50 | 100 | 100 | 100 |
| Fluence rate [mW/cm2] | | | － | 25 | 25 | 25 | 100 | 300 |
| Study  group | | | 1 | 2 | 3 | 4 | 5 | 6 |
